# Dimensional Nanofillers in Mixed Matrix Membranes for Pervaporation Separations: A Review

**DOI:** 10.3390/membranes10090193

**Published:** 2020-08-19

**Authors:** Guang Yang, Zongli Xie, Marlene Cran, Chunrui Wu, Stephen Gray

**Affiliations:** 1 Institute for Sustainable Industries and Liveable Cities, Victoria University, P.O. Box 14428, Melbourne, VIC 8001, Australia; guang.yang@csiro.au (G.Y.); marlene.cran@vu.edu.au (M.C.); 2 CSIRO Manufacturing, Private bag 10, Clayton South, VIC 3169, Australia; 3 State Key Laboratory of Separation Membranes and Membrane Processes, Institute of Biological and Chemical Engineering, Tianjin Polytechnic University, Tianjin 300387, China; wuchunrui@tjpu.edu.cn

**Keywords:** pervaporation, nanofiller, polymer, solvent dehydration, desalination, solvent recovery

## Abstract

Pervaporation (PV) has been an intriguing membrane technology for separating liquid mixtures since its commercialization in the 1980s. The design of highly permselective materials used in this respect has made significant improvements in separation properties, such as selectivity, permeability, and long-term stability. Mixed-matrix membranes (MMMs), featuring inorganic fillers dispersed in a polymer matrix to form an organic–inorganic hybrid, have opened up a new avenue to facilely obtain high-performance PV membranes. The combination of inorganic fillers in a polymer matrix endows high flexibility in designing the required separation properties of the membranes, in which various fillers provide specific functions correlated to the separation process. This review discusses recent advances in the use of nanofillers in PV MMMs categorized by dimensions including zero-, one-, two- and three-dimensional nanomaterials. Furthermore, the impact of the nanofillers on the polymer matrix is described to provide in-depth understanding of the structure–performance relationship. Finally, the applications of nanofillers in MMMs for PV separation are summarized.

## 1. Introduction

With the rapid development over the past 40 years, membrane technologies have been increasingly deployed for industrial processes, including wastewater treatment [1], desalination [2], organic solvent dehydration [3] and gas separation (CO_2_ removal, H_2_ isolation, O_2_ enrichment) [4]. Pervaporation (PV) is one of the membrane separation processes that applies a non-porous barrier, allowing the permselective transport of guest molecules between the feed (liquid mixture) and permeate side [5]. Different from conventional membrane filtration processes, such as microfiltration [6], ultrafiltration [7], nanofiltration [8] and reverse osmosis [9], the solutes in the PV processes go through a phase transition from liquid to vapor across the membrane [10]. Therefore, PV is inherently appropriate for volatile/volatile or volatile/non-volatile separation. PV has been widely employed in solvent dehydration and organic–organic separation since the first industrial apparatus for ethanol dehydration was designed by GFT (DeltaMem AG) in Brazil (1984) [11,12]. In recent years, desalination by PV has received great research interest due to its capacity to cope with hypersaline water, whereas huge energy is required to overcome the osmotic pressure in typical reverse osmosis processes [13,14,15]. In general, polymeric membranes are most studied and applied in PV processes as they are inexpensive, easy to process and scalable [16]. Poly (vinyl alcohol), chitosan (CS) and sodium alginate (NaAlg) are popular as membrane materials for dehydration of a range of alcohols [16]. For instance, the GFT PVA composite membrane was reported to exhibit a water flux of ~0.14 kg m^-2^ h^-1^ and ~20 wt% ethanol in permeate when the feed was a binary ethanol–water system (50 wt% ethanol) at 35 °C and ~400 Pa of downstream pressure [17]. However, the polymeric membranes are proven to suffer from the trade-off between selectivity and permeability, and also low chemical and thermal resistance in certain applications. 

Mixed matrix membranes (MMMs) or hybrid membranes are a class of heterogeneous polymer-based membranes consisting of a discrete inorganic phase, a continuous polymer phase and an organic–inorganic interphase [18]. The inorganic phase usually takes the form of micro- and nano-level materials, which can also be referred to as fillers or additives. The concept of MMMs, first originated when zeolite 5A molecular sieves, were incorporated into a polydimethyl siloxane (PDMS) matrix for gas separations in 1973 [19,20]. In general, inorganic materials’ own merits include resistance to harsh chemical environments, good rigidity and high thermal stability [21]. These properties show attractive promise for long-term membrane operation. By incorporating inorganic fillers into the polymer matrices, MMMs can overcome the limitations of inorganic membranes while inheriting various perm-selective characteristics of inorganic fillers and polymers, thus combining the ease of processing polymer membranes while exhibiting great potential to surpass the trade-off encountered with neat polymer membranes [22]. The techniques for the preparation of MMMs are similar to those used for the preparation of polymeric membranes but with the addition of inorganic fillers. The most common approach is via the physically blending of the polymer and the filler to form a dope solution followed by solution-casting on a substrate. For instance, Qian et al. mixed CS and graphene oxide (GO) uniformly in aqueous environment and cast the solution on a glass plate to form an MMM for PV desalination [23]. The sol-gel process is another well-known method to prepare nanoparticles (NPs) in situ by hydrolysis and the condensation of inorganic precursors (typically metal alkoxides) in the polymer matrix. MMMs containing nanosized silica and titanium dioxide (TiO_2_) derived from a series of precursors, such as tetraethoxysilane, 3-glycidyloxypropyltrimethoxysilane, tetrabutyl titanate and titanium tetrachloride, have been widely reported with excellent dispersion state [24,25,26,27]. Recently, unconventional methods including in situ polymerization of a polymer matrix or synthesis of fillers have also been applied in the development of PV MMMs. Mao et al. used metal organic framework (MOF) precursors to generate zeolitic imidazolate framework (ZIF) nanoparticles in a PDMS matrix in situ and obtained simultaneous enhancement in permeability and selectivity for ethanol dehydration [28]. Bai and co-workers synthesized ethylene-vinyl acetate based MMM by the in situ polymerization of hyperbranched polysiloxane [29] with the MMM exhibiting the enhanced selective transport of ethyl acetate while rejecting water, which was due to improved hydrophobicity after the addition of polysiloxane.

**Figure 1:**
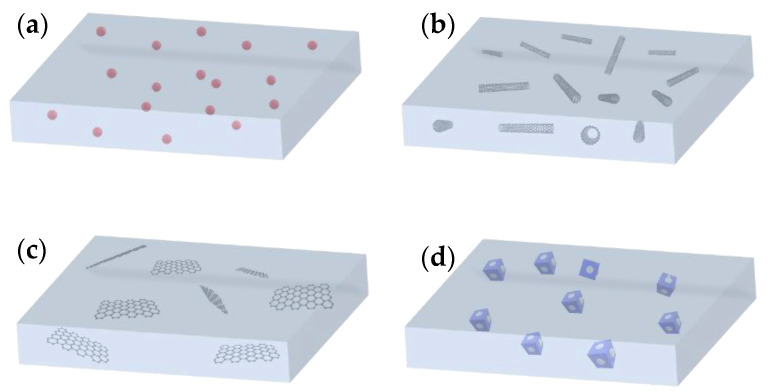
MMMs with different dimensional nanofillers; (**a**) zero-dimensional nanomaterial filled MMM (nanoparticles); (**b**) one-dimensional nanomaterial filled MMM (carbon nanotube for example); (**c**) two-dimensional nanomaterial filled MMM (graphene oxide nanosheet for example); (**d**) three-dimensional nanomaterial filled MMM (microporous nanomaterials).

In the case of the preparation of MMMs, the compatibility of filler/polymer and the homogeneous dispersion of the fillers are the most critical issues. To date, multifarious nanofillers have been incorporated into polymer matrices to form MMMs and, in general, nanofillers can be divided into four categories—i.e., zero-, one-, two- and three-dimensional nanomaterials—and the MMMs with various dimensional nanofillers can be obtained accordingly (Figure 1). NPs, such as gold, silver, zinc, or metal oxides (usually 1–50 nm), in the membranes are typical representatives for zero-dimensional nanofillers [30,31,32]. One-dimensional nanotubes, nanorods, nanowires and nanofibers are needle-like shaped nanomaterials whereas two-dimensional nanomaterials are thin nanosheets that have only one external dimension in the nanoscale [33,34,35]. The area of two-dimensional nanomaterials can be up to a few square microns, typically far exceeding their thicknesses [36]. Nanoporous materials, such as silicalites, zeolites and MOFs with polycrystalline structures can be considered as examples of three-dimensional nanofillers [37,38]. It should be noted that bulk NPs, bundles of one-dimensional materials and multi-nanolayers, can also be considered as three-dimensional nanomaterials, thus affording tunable properties of nanofillers based on their dispersion state. Owing to the abundance of dimensional nanomaterials, as well as their specific chemistry, different combinations between nanofillers and polymers give rise to great flexibility in the design of hybrid membrane structures. This flexibility is highly associated with the separation performance and therefore arouses extensive research interest.

Herein, we review the current developments of PV MMMs with a special focus on the dimensional nanofillers that have been investigated to date. The exploitation of nanomaterials from zero to three dimensions is systematically discussed, followed by the impact of nanofillers on the polymer matrix to tentatively unveil the structure–performance relationship for PV processes. Recent progress on the applications of PV MMMs is also discussed and finally, the future perspectives for the rational design of organic–inorganic MMMs is proposed.

## 2. Nanofillers Used in MMMs

To date, various combinations between nanofillers and polymers have been applied for PV separation processes. Typical nanofillers with different dimensions, such as zero-dimensional nanoparticles, one-dimensional nanotubes, two-dimensional nanosheets and three-dimensional framework nanomaterials have been incorporated into polymer matrices, and these are listed in Table 1. The nanofillers are systematically discussed in terms of their dimensions in 2.1–2.4. For the evaluation of PV membrane performances, the throughput and separation efficiency are considered in terms of permeation flux and separation factor or permeance and selectivity. In particular, permeance and selectivity are preferred to evaluate the membrane performance since they are material characteristics that reveal the intrinsic separation properties of the membrane [39]. However, permeance and separation factor are not always reported and cannot always be calculated from previous work as often essential data are not reported. 

### 2.1. Zero-Dimensional Nanofillers

**Figure 2:**
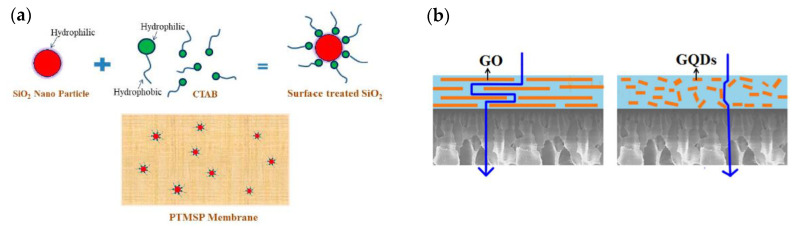
(**a**) Surface modification of silica NPs and the PTMSP/silica MMM. Reproduced with permission from [40], published by MDPI, 2011. (**b**) Comparison between permeation of GO filled MMM and GOQDs filled MMM. Reproduced with permission from [43], published by Elsevier, 2018.

The most well-known zero-dimensional nanomaterials are nearly spherical NPs encompassing noble metal NPs, metal oxide NPs and metalloid derived NPs [59]. When incorporated into a polymer matrix, the nanoscale NPs possess high specific area with abundant functional groups, enabling good interfacial compatibility with the polymer and thus a uniform dispersion state. Silica NPs are one such cost-effective nanomaterials with low toxicity and, in one example, Xie et al. used tetraethoxysilane as a precursor to prepare silica NPs (<10 nm) via a sol-gel process [24]. The obtained PVA based MMM with highly dispersed silica NPs (up to 10 wt% with respect to PVA) exhibited enhanced separation performance in terms of water permeation flux and salt rejection as compared with the control sample without silica. In another example, due to the existence of silanol groups on the surface, hydrophilic silica NPs were modified by hexadecyltrimethylammonium bromide (CTAB) to introduce hydrophobicity, as shown in Figure 2a. The CTAB modified silica was incorporated into poly(1-trimethylsilyl-1-propyne) (PTMSP) for the selective removal of 1-butanol from aqueous solutions. The resulting PTMSP/CTAB-silica membrane obtained significantly increased butanol diffusivity (15% higher than neat PTMSP membrane) with a separation factor of 131 [40]. A range of metal based NPs, Fe_3_O_4_, TiO_2_, Ag, etc. have also been successfully investigated as nanofillers to produce high-performance MMMs [26,41,60].

Carbon-based zero-dimensional nanomaterials, including fullerene, graphene quantum dots (GQDs) and graphene oxide quantum dots (GOQDs) are also of interest in modifying the separation performance of MMMs [61]. In particular, GQDs are emerging nanomaterials with a single or a-few-layered graphene quasispherical structures of diameters below 100 nm. Owing to the small sizes, GQDs were used to cover the structural defects in the reduced graphene oxide (rGO) incorporated alginate (Alg) membrane [42]. The resulting Alg-rGO-GQD MMM showed great potential in separating small molecules (methanol/water) with a permeation flux of 2323 ± 45 g m^−2^ h^−1^ and water concentration in permeate of 92.7 ± 0.03% at 70 °C. GOQDs inherit the sp2 carbon monolayer structure, as with that in GQDs, but with additional oxygen-containing groups, suitable to form MMMs with a hydrophilic polymer. Jiang and coworkers utilized GOQDs with an average lateral dimension of around 3.9 nm as nanofillers to prepare NaAlg-based MMM [43]. The dehydration of ethanol was performed on the NaAlg-GOQDs membrane with 60% higher permeation flux than that of neat the NaAlg membrane, which could be attributed to additional shorter and less tortuous transport pathways provided by GOQDs, as shown in Figure 2b. Although zero-dimensional nanomaterials can modify the physicochemical properties and enhance the separation performances of MMMs, they are impermeable and that means they may not provide additional transport channels for solutes within the membranes, which may not be beneficial for further flux increase upon higher loading of zero-dimensional nanomaterials.

### 2.2. One-Dimensional Nanofillers

Since the discovery of carbon nanotubes (CNTs) in 1991, one-dimensional nanostructured materials have gained increasing attention as the building blocks for membrane separation applications [62]. Different from zero-dimensional materials, one-dimensional nanomaterials can be assembled as a separation membrane or substrate. Livingston and coworkers synthesized sub-10 nm thick polyamide nanofilms on a well-constructed cadmium hydroxide nanostrand layer and reported that the obtained polyamide membrane exhibited ultrafast organic solvent nanofiltration [63]. Hu et al. prepared a CNT-based membrane capable of oil-in-water separation with a maximum flux up to 35,890 L m^−2^ h^−1^ bar^−1^ [64]. The interpenetrated and densely packed structure of the above-mentioned one-dimensional materials enables the formation of nanopores, allowing the transport of molecules. However, when used as nanofillers, one-dimensional materials need to be dispersed homogeneously to avoid agglomeration and nonselective voids.

Yang et al. compared the separation performances of PVA/CNT and PVA/functionalized CNT MMMs during PV desalination [44]. The results showed that carboxyl functionalized CNTs had good interfacial compatibility, whereas the nonfunctionalized CNTs intertwined significantly in the polymer matrix. The corresponding separation performances suggested that the excellent interfacial compatibility and subsequent good dispersion of functionalized CNTs enhanced the transport of water while effectively blocking ions, showing great potential for the practical application of PV for desalination. Furthermore, hydrophilic modifications of CNTs by NPs (Ag, TiO_2_ and Fe_3_O_4_) and polymer molecules have also been conducted [65,66] whereby these approaches can effectively provide steric barriers to suppress van der Waals forces between CNTs. For instance, Gao et al. decorated CNTs using Fe_3_O_4_ NPs and incorporated the Fe_3_O_4_/CNT into a NaAlg matrix [45]. The resulting MMMs exhibited high water permeation flux due to the fast transporting micro-channels of CNTs. Jiang and coworkers employed chitosan-wrapped CNTs as nanofillers in a PVA based MMM [67] for the PV separation of benzene/cyclohexane and demonstrated simultaneous increase in permeation flux and separation factor compared with pure PVA membrane. Molecular dynamic simulation suggested that the enhancements were attributed to preferential affinity of CNTs with benzene and the nanostructural change of the PVA matrix.

**Figure 3:**
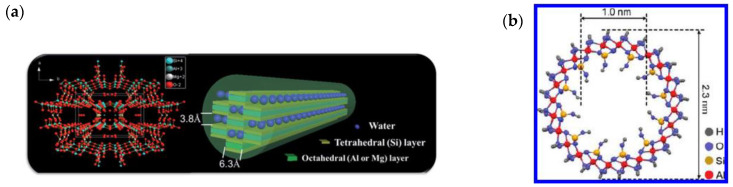
(**a**) Structure of attapulgite nanorod. Reproduced with permission from [46], published by Royal Society of Chemistry, 2016. (**b**) Aluminosilicate single-walled nanotube. Reproduced with permission from [48], published by American Chemical Society, 2012.

In addition to CNTs, metal oxide nanotubes, attapulgite nanorods, titanate nanotubes and aluminosilicate nanotubes have also been applied in MMMs for PV separations [47,48] and the structures of nanorods and nanotubes are shown in Figure 3. For example, Xing et al. incorporated natural hydrophilic attapulgite nanorods into a NaAlg matrix [46]. Water inside the membrane formed hydration layers along the nanorods due to plentiful -OH groups. As a result, the hybrid membrane showed the outstanding dehydration of ethanol with good structural stability. Different from CNTs, aluminosilicate nanotubes present unique interior hydrophilicity due to the existence of inner surface silanol groups. The abundant outer surface groups of aluminosilicate nanotubes render a high affinity with polymer matrices, allowing for the individual dispersion of nanotubes without agglomeration. Nair and coworkers prepared PVA/aluminosilicate nanotube MMMs for ethanol dehydration [48] and showed that the nanofiller loading could be up to 40 vol%, which overcame the limitation of CNT loading in PVA (usually 0.5~3 wt%). The resulting membrane was subjected to a PV process for ethanol dehydration with water throughput that was ~200% higher than the pure PVA (similar thickness in the range of 40–100 μm) membrane and decrease in separation factor from 58 (pure PVA) to 35 (PVA/aluminosilicate nanotubes). One-dimensional nanofillers, such as CNTs and aluminosilicate nanotubes are promising for the provision of additional transport pathways due to their inherent nano-scale fast transport channels. A key factor to realizing their potential for fast transport is their uniform dispersion in the polymer matrix. As investigated in previous studies [44,68], the agglomeration of CNTs increased the water flux but resulted in the decrease in membrane selectivity. Interfacial compatibility is required to achieve good dispersion. However, the chemical modification of CNTs to functionalize their surface can lead to the generation of defects [69], thereby restraining the potential to facilitate rapid mass transport. Suitable modification methods are sought to overcome this drawback to realize the potential of one-dimensional nanofillers.

### 2.3. Two-Dimensional Nanofillers

**Figure 4:**
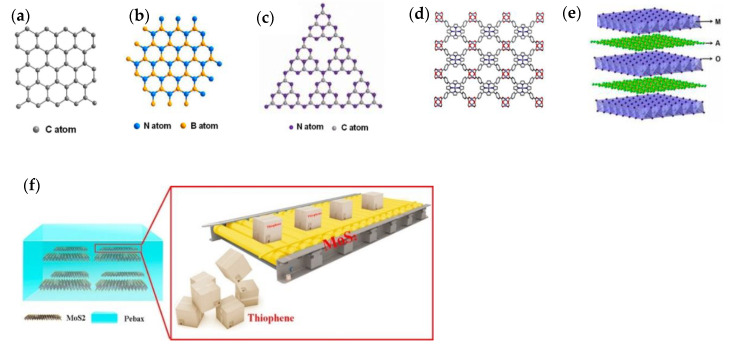
Structures of (**a**) graphene, and (**b**) hexagonal boron nitride, (**c**) s-triazine structure models of g-C_3_N_4_, (**d**) MOF nanosheet and (**e**) layered double hydroxides. Reproduced with permission from [71], published by American Chemical Society, 2017. (**f**) MoS_2_ in Pebax as transport facilitator for thiophene. Reproduced with permission from [52], published by Elsevier, 2018.

Over the last decade, two-dimensional materials with thicknesses from single to a few atoms have been rapidly developed in the context of electrocatalysis, batteries, supercapacitors, solar cells, photocatalysis and sensors [70,71]. In the two-dimensional nanomaterial family, graphene and graphene oxide are exemplary models owing to the high specific surface area, Young’s modulus, electrical and thermal conductivities. Emerging members are MXenes, graphitic carbon nitride (g-C_3_N_4_), layered double hydroxides, hexagonal boron nitride, two-dimensional MOF nanosheets, covalent–organic frameworks (COFs) nanosheets, inorganic perovskite nanosheets and more, as shown in Figure 4. Two-dimensional materials can be engineered to provide sub-nanometer channels by stacking layers in parallel. However, the transport through the in-plane and out-of-plane directions is tortuous and this prolongs the molecule permeation paths across the membrane. One strategy to address this is by creating vacant nanopores on a single nanosheet, which shortcuts the in-plane transport. In this vein, the control of high-quality nanopores, with characteristics such as uniform size, suitable geometric shape and enough density on the nanosheets, is very challenging for their large-scale application in two-dimensional membranes.

Alternatively, polymer/nanosheet MMMs are facile to prepare with greater control without significant compromise in separation performances when compared with pure two-dimensional laminar membranes. Integrating GO with various polymers has been popular and proved to be an efficient method to improve the separation performance in different membrane applications, including ultrafiltration, nanofiltration, reverse osmosis, or forward osmosis. For PV separations, GO has been added into various polymer matrices, such as PVA [72], NaAlg [73], polyimide [49], polyamide [74] and polyetherimide [75]. Benefiting from the negatively-charged surface and strong hydrophilicity, MMMs containing GO usually exhibit elevated water permeation and enhanced rejection of ions or organic solvents. For instance, Qian et al. investigated the effect of GO on CS based MMM in the PV desalination process [23]. It was concluded that both water permeability and water/salt selectivity increased with growing GO content. The incorporation of GO could effectively retard NaCl diffusion in the membranes, resulting in a reduced diffusion coefficient of two orders of magnitude lower than water.

Recently, two-dimensional MXene nanosheets, g-C_3_N_4_ nanosheets and molybdenum disulfide (MoS_2_) nanosheets have been attempted as nanofillers for the use of CS [50], NaAlg [51] and Pebax [52] based MMMs, respectively. These nanosheet MMMs exhibited improved water permeability and selectivity, which was associated with the special chemistry of the two-dimensional materials. In the water/ethanol separation process using g-C_3_N_4_/NaAlg MMMs, well ordered channels for water transport in the membrane were produced by horizontally aligned g-C_3_N_4_ nanosheets enabling an additional sieving effect, resulting in excellent separation performance exceeding other NaAlg based membranes. For the separation of thiophene/n-octane mixture (Figure 4f), MoS_2_ showed moderate binding energy with thiophene and rendered facilitated transport pathways on the basal plane for fast thiophene diffusion. The Pebax/MoS_2_ MMM exhibited a 22.27% and 65.94% increase in permeation flux and enrichment factor, respectively, as compared with those of the pure Pebax membrane. Two-dimensional nanosheets possess many merits as nanofillers in MMMs due to their unique chemistry and ultrathin thicknesses. However, the lateral sizes of some two-dimensional materials are relatively large, being on the micron-scale [76], which may limit the fabrication of MMMs if the membrane thickness is thinner than the lateral size.

### 2.4. Three-Dimensional Nanofillers

Zeolite frameworks are crystalline silicates or alumino-silicates, possessing regular-shaped nanopores smaller than 2 nm [38]. Commonly, the SiO_4_ or A104 tetrahedrons are linked by sharing oxygen atoms to form cavities or cages, thus rendering molecular sieving properties [12]. Interestingly, the properties of nanopores, such as hydrophilic, hydrophobic, acid, or basic properties can be tuned by Si/Al stoichometry. For example, in PV MMMs, hydrophobic zeolite (ZSM-5) was added to PDMS for the selective removal of ethanol from water [53] and the zeolite loading of up to 40 wt% was found to have a significant effect on separation performance, achieving a high selectivity of 14. Mohammadi and coworkers studied polyimide/zeolite 4A MMM for hydrophilic PV [54]. The results manifested that both water permeation flux and selectivity were increased by zeolite 4A, reaching a separation factor of 8991 and a permeation flux of 0.018 kg m^−2^ h^−1^ at 30 °C.

**Figure 5:**
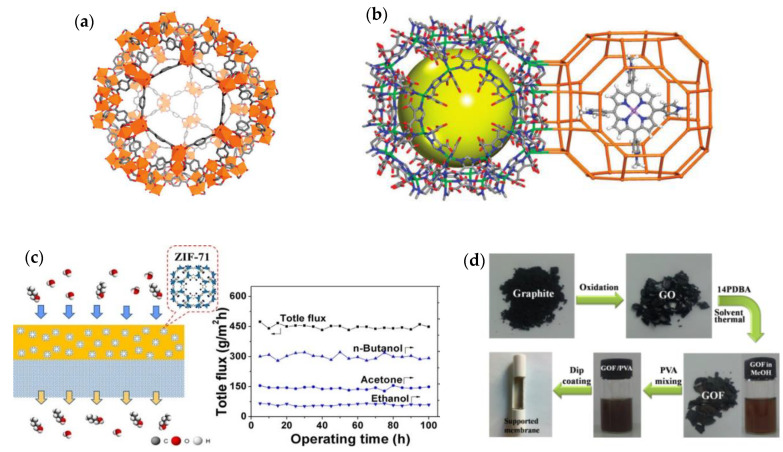
(**a**) Pore structure of MIL-101, (**b**) pore structure of rho-ZMOF (left) and encapsulated [H_2_TMPyP]^4+^ porphyrin in RHO-ZMOF a-cage (right). Reproduced with permission from [70], published by Royal Society of Chemistry, 2009. (**c**) Mass transport of ZIF-71 incorporated membrane and its performance. Reproduced with permission from [55], published by Elsevier, 2013. (**d**) GOF/PVA MMM fabrication. Reproduced with permission from [58], published by Royal Society of Chemistry, 2015.

As a structural analogue to zeolites, MOFs feature remarkably high surface area, large pore volume and uniform cavity size [77]. However, they differ in chemical compositions whereby zeolites are inorganic materials and the MOFs are organic–inorganic hybrids containing metal ions or clusters coordinated to organic ligands, as shown in Figure 5. Consequently, MOFs impart greater diversity in chemistry than zeolites and also have potential as nanofillers in MMMs. So far, various MOFs, including the MIL type (Materials Institute Lavoisier), ZIF type (zeolitic imidazolate frameworks), UiO type (Universitetet i Oslo) and HKUST type (Hong Kong University of Science and Technology), have been applied in MMMs for PV with serious consideration of solvent stability. For example, Liu et al. synthesized ZIF-71 with average size of 1 μm and used polyether-block-amide (PEBA) as the polymer matrix for butanol recovery from the fermentation of acetone/butanol/ethanol [55]. The resulting MMM showed promising separation performance for practical butanol recovery due to excellent compatibility between ZIF-71 and PEBA. Chung and coworkers designed UiO-66/polyimide MMM for alcohol dehydration [78] and after the addition of UiO-66, both free-volume radius and fractional free volume were modified, favoring the water transport through the membrane. The MMM with 30 wt% UiO-66 loading had high water flux of 0.329 kg m^−2^ h^−1^ and a separation factor of 2209 for isopropanol/water, which were superior to the other polyimide based MMMs.

In addition to zeolites and MOFs, one- or two-dimensional nanomaterials can also be manipulated into complex and hierarchical architectures, granting porosity and similar properties of three-dimensional nanomaterials. For example, polyhedral oligomeric silsesquioxane (POSS) [79] was built up by a silicon–oxygen framework with a formulated three-dimensional “cage-shaped”. Due to the organic groups in the framework, POSS is highly compatible with the polymer matrices and can thus be employed as nanofillers to improve PV performance. Furthermore, the other three dimensional nanomaterials, such as sodium montmorillonite (Na^+^-MMT), carbon molecule sieve (CMS) and graphene oxide framework (GOF) have also been investigated to provide barrier properties in MMMs [56,57]. In one example, stable and uniform interlayer-spacing significantly improved the molecular sieving property of benzene diboronic acid-linked GOF [58]. As a consequence, the PVA/GOF MMM excluded the diffusion of solvent (≥C3) during alcohol dehydration, outperforming other polymeric and inorganic PV membranes in terms of dehydration of propanol and butanol. Overall, three-dimensional nanofillers, such as MOFs, exhibit excellent molecule sieving properties, which is ideal for membrane separations. However, the stability of MOFs and other three-dimensional nanomaterials during long-term operation is yet to proven for their practical application.

## 3. Effect of Nanofillers on Polymer Matrix

Upon the incorporation of nanofillers, the physicochemical properties of resulting hybrid materials are commonly found to be altered when compared with the pristine polymeric network [22]. The changes occurring in the hybrid materials can be associated with the intricate organic and inorganic interactions both physically and covalently [20,80]. A deep insight into the effect of nanofillers on the polymer matrix is required to link the membrane structure and separation performance for rational MMM design. In gas separation, there are several models describing separation processes [81]. The solution–diffusion model is the most well-known and used model for PV processes, but there are few modelling studies focusing on the effects of nanofillers for PV MMM performance. To date, several key traits in terms of surface, thermal and chemical properties, as well as membrane morphology, have been demonstrated as playing important roles in separation process.

### 3.1. Effect of Nanofillers on Morphology of MMMS

**Figure 6:**
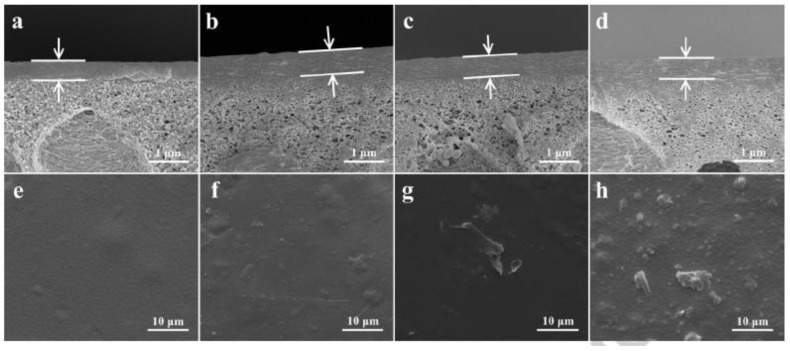
SEM cross-section images of (**a**) pure NaAlg on PAN substrate; (**b**) (**c**) and (**d**) MMMs containing g-C_3_N_4_ content 2, 3 and 5 wt% with respect to NaAlg. (**e**–**h**) Corresponding surface images of (**a**) to (**d**), respectively. Reproduced with permission from [51], published by Elsevier, 2015.

The aggregation of nanofillers and non-expected interfacial voids are long-standing issues that impact the morphology of MMMs, usually resulting in concave–convex appearance when observed using scanning electron microscope (SEM) imaging. Due to the large area-to-volume ratio and geometric characteristics, nanoscale materials are highly inclined to aggregate. This trend increases with the concentration of nanofillers in the polymer due to the increasing probability to contact. Consequently, such a concentration-dependent phenomenon has been widely seen in PV MMMs when nanomaterials, such as TiO_2_, GO, g-C_3_N_4_ and ZIF, are used as fillers. As shown in Figure 6, taking g-C_3_N_4_ filled NaAlg MMMs on polyacrylonitrile (PAN) support, for example, both the cross-section and surface images indicated elevated aggregate formation by increasing the loading of nanosheets (from 1 wt% to 5 wt%), as compared with the pure NaAlg [51]. Correspondingly, the surface topography of MMMs also showed related changes, either smoother or rougher after the incorporation of nanofillers. Xie and coworkers demonstrated that the surface roughness of silica/PVA MMMs increased with the concentration of silica, which corresponded to Si-O-Si self-congregation reaction [82]. Conversely, the number of indents and protuberances on the surface of the GOQDs incorporated NaAlg membrane was less than that of the neat NaAlg membrane, resulting in an average roughness from 10.36 nm to 6.94 nm [43]. Such observations can be attributed to the highly affinitive interactions (hydrogen bonding) between GOQDs and NaAlg functional groups during membrane formation.

### 3.2. Effect of Nanofillers on Free Volume Properties

In polymeric membranes, free volume is generated by the unoccupied space that exists between polymer chains. It is an inherent property that can be regarded as the channels whereby molecules diffuse through the membrane [83]. Therefore, the free volume property is highly suspected to have a significant impact on membrane permeability and selectively. Zhao et al. showed that the fractional free volume of a zwitterionic GO/NaAlg MMM became smaller with a subsequently larger pore size when the zwitterionic GO content was increased [84]. As a result, a higher permeability and a greater separation factor were obtained for the dehydration of ethanol. In addition, the effect of heat treatment on the free volume of PVA/silica MMM was systematically investigated [85] and enhanced selectivity was observed with a sacrifice in water permeation flux due to the more compact structure induced by heat treatment. Similarly, the POSS filled polyimide exhibited a diminished average pore size and fractional free volume, giving rise to increased membrane selectivity but decreased permeation flux [79].

### 3.3. Effect of Nanofillers on Swelling

Polymer swelling usually takes place via defined solvent molecule diffusion into the polymer matrix resulting in swollen gel formation [86]. Due to the existence of intermolecular interactions, such as crosslinks, crystallites or strong hydrogen bonding, the dissolution of the polymer can be restrained [87]. However, these interactions can significantly affect the membrane structure and thus the separation performance. Nanofillers in the polymer matrix, either physically doped or covalently linked, exert a specific impact on the swelling behavior of MMMs. Jin and coworkers developed a hydrophilic MOF-801/CS (physically doped) MMM for ethanol dehydration [88]. Despite the fact that MOF-801 with high porosity may generate the additional solvent adsorption, the strong interfacial hydrogen bonds with CS prevented the movement of CS chains and thereby offset the solvent adsorption in the polymer matrix. The swelling degree of MMMs showed negligible influence upon the introduction of MOF-801, as compared with the CS membrane. PVA/GOQDs MMMs have also shown higher swelling resistance due to additional cross-linking reactive groups between –COOH from GOQDs and -OH from PVA [89].

### 3.4. Effect of Nanofillers on Surface Properties

Depending on the targeted application, either hydrophilic PV membrane (for desalination and solvent dehydration) or organophilic PV membranes (for solvent recovery and organic–organic separation) are desired. It is well-known that hydrophilic membranes have an affinity for water molecules and are more resistant to membrane fouling than hydrophobic membranes. As such, it is expected that the introduction of nanofillers contributes to the surface hydrophilicity or hydrophobicity by facilitating the adsorption of a higher concentration of solutes adsorbed on the membrane surface, resulting in a greater concentration gradient across the membrane for molecule transport.

In one example, MOF consisting of RHO-[Zn(eim)_2_] (MAF-6) was incorporated into PDMS for organophilic PV [90]. Benefiting from the superhydrophobicity of MAF-6, the water contact angle increased from 95° (pure PDMS) to 119.8° when the content of MAF-6 was 15 wt%. The corresponding MMM showed an ethanol flux of 1.5 times and a separation factor of 2.3 times that of the PDMS pristine membrane. For hydrophilic PV membranes, the water contact angle of CS/GO MMM was smaller than the pure CS membrane, owing to the oxygen-containing groups on GO nanosheets [23]. In contrast, when hydrophobic nanofillers were added into hydrophilic polymer matrix, the water contact angle usually increased, as occurred in ZIF-8 and CNT incorporated NaAlg and PVA membranes, respectively [44,91]. As a result, the permeation flux was enhanced but with a subsequent reduction in the membrane’s selectivity, which can be attributed to the incompatibility at the interface between nanofillers and polymer matrix and non-selective void formation.

### 3.5. Effect of Nanofillers on Thermal Properties

The high thermal stability of the membrane is favorable for the PV processes as the feed solution is often pre-heated to the required temperature before routing to the PV module [10]. If the polymer is highly susceptible to temperature—e.g., low glass transition and melting temperature—the molecular structure of the membrane can be modified with the temperature of the feed, which can result in the unexpected separation of molecules. In general, polymer chains exhibit elevated mobility with temperature, which allows for the faster but nonselective permeation of molecules. Conversely, inorganic materials exhibit high thermal stability with the capacity to withstand massive heat stress. Organic–inorganic hybrids impart combined thermal properties, whereby the inorganic filler can enhance the thermal stability as compared with the pristine polymer [92]. In such a multiphase system containing a polymer continuous phase, dispersed inorganic phase and organic–inorganic interphase, the nanofillers may endow a heat barrier effect upon the uniform dispersion, which restrains the heat transfer within the polymer matrix [93].

Choudhari et al. used thermo gravimetric analysis to compare the thermal degradation behaviors of CS and Na^+^-MMT clay filled CS membranes [94]. The degradation and deacetylation of the CS membrane occurred over the temperature range of 200–450 °C. At the same point of 50% weight loss, the temperature of Na^+^-MMT clay filled CS membrane was around 5–30 °C higher than that of the CS membrane. That could be due to factors such as good heat barrier properties of Na^+^-MMT clay, and strong interaction between polymer and nanofillers. Penkova and coworkers investigated the effect of carboxyfullerene on the glass transition temperature (T_g_) of the PVA based MMMs using differential scanning calorimetry [95]. Compared with the neat PVA, the PVA filled with carboxyfullerene had a stiffened structure with increased T_g_ that was 10 °C higher. This result showed restricted polymer chain motion induced by carboxyfullerene, possibility due to covalent linkage formation between PVA and carboxyfullerene.

### 3.6. Effect of Nanofillers on Polymer Crystallinity

Generally, polymer crystallization is constructed by rigid and aligned chain packing, forming a highly ordered region that can affect physicochemical properties, involving mechanical stability, melting point and transparency [96]. In the case of polymer membranes, the crystallites restrict the permeation of molecules due to the compactly arranged structure and a lack of polymer chain mobility. Most polymers are semi-crystalline, composed of both amorphous and crystalline phases where the amorphous phase is formed by disordered polymer chains that are dispersed in the polymer matrix and physically linked by the crystalline regions. For polymeric membranes, the permeation of molecules occurs via the transport through the amorphous phase, whereas the crystalline phase acts as an impermeable barrier. It is, therefore, possible to design membrane selectivity by controlling polymer crystallization. For example, a previous study provided a favorable membrane design for ultrafast organic solvent nanofiltration by preparing amorphous conjugated polymers with rigid backbones [97]. The highly rigid networks provided massive interconnected voids, which enabled high stability in organic solvents and the fast transport of small molecules. Inspired by that, amorphous but rigid polymer composition may be beneficial for high-performance separation. Organic–inorganic hybrid materials have the potential to be used for realizing such a membrane design as the incorporation of inorganic nanofillers can tune the crystallinity of the polymer by either interrupting the chain packing or by acting as nucleating agents to enhance crystallization [98].

Cao et al. added GO into the NaAlg matrix for ethanol dehydration [99]. The hydrogen bonding between nanofillers and NaAlg significantly disrupted the chain packing, leading to a drop in crystallinity when the content of GO was low. However, the crystallinity of NaAlg began to increase when the GO loading was over 1.2 wt%, which was considered to be due to the nucleating effect caused by nanosheets as the dominant factor. The addition of ZIF-8 into a PVA matrix increased the crystallization degree because ZIF-8 provided a nucleation site for PVA chains [100]. On the contrary, the agglomerates of ZIF-8 particles decreased the crystallinity of the PVA-based MMM when its loading was relatively high. When used as crosslinkers, increasing the content of silica nanoparticles continuously decreased the crystallinity of the PVA matrix. That was attributed to the covalent linkage formation between polymer chains and silica, resulting in the destruction of chain alignment and then a crosslinked network for the PV desalination process.

### 3.7. Effect of Nanofillers on Chemical Properties

**Figure 7:**
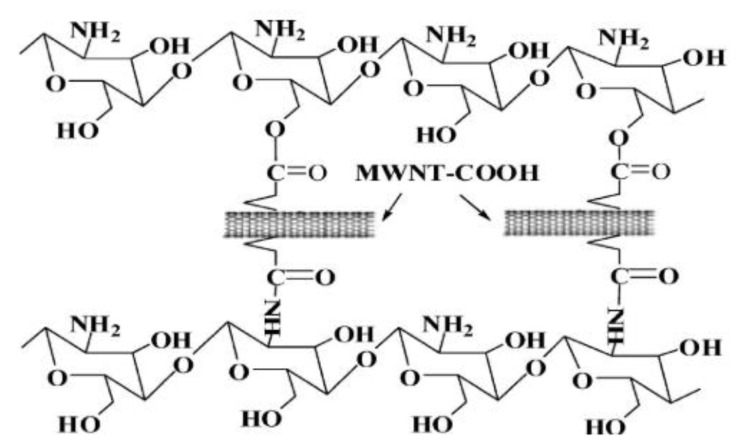
Interfacial linkage in carboxylic CNTs with CS. Reproduced with permission from [105], published by American Chemical Society, 2010.

For some polymers, the intrinsic hydrophilicity or hydrophobicity can impart high permeability for specific affinitive solutes. However, uncontrolled swelling behavior necessitates chemical modification of the polymer chains to render them of adequate ability for membrane separations. In this respect, crosslinking is an effective step to manipulate the polymer for membrane synthesis, which enables a compact and covalently linked polymer network with good chemical and thermal stability [101]. Crosslinking is based on the chemical reaction of functional groups between the polymer and crosslinker, which is typically a relatively small molecule with multi reactive groups. Likewise, the functional groups from nanofillers can also form covalent linkage or ionic complex (using metal ion as crosslinker) with polymer chains, thereby connecting or grafting the polymer chains. For instance, silica from different precursors, including γ-glycidyloxypropyltrimethoxysilane, phenyltriethoxysilane, γ-aminopropyl-triethoxysilane, tetraethoxysilane, diethoxydiphenylsilane, etc., have been widely applied in PVA as reactive nanofillers [25,102,103,104]. The hydrolysis and self-condensation processes produced silica nanoparticles with substantial silanol groups, which can react with hydroxyl groups to form C–O–Si bonds. In addition, carbon based nanomaterials, such as carboxylic CNTs and GO, have been used to crosslink CS chains on the basis of an esterification reaction between –COOH and –OH [105] as shown in Figure 7. The ionic crosslinking of NaAlg has also been successful using multivalent ions, including Ca^2+,^ Zn^2+^, Mn^2+^, Fe^2+^, Co^2+^ and Al^3+^ [106]. The salinization of the carboxyl groups from NaAlg was investigated by Fourier transform-infrared spectroscopy, revealing the crosslinking mechanism within the polymer matrix.

Immobilizing nanofillers in the polymer matrix via non-covalent interactions, such as hydrogen bonding and physical interactions (interfacial adhesion and mechanical interlocking) is also a widely implemented strategy. In this case, the nanofiller can retain its chemistry, exhibiting specific functions during subsequent separation processes. Aminabhavi and coworkers dispersed aluminum-rich zeolite in NaAlg [107]. The NaAlg chains were crosslinked with glutaraldehyde and the zeolite particles were physically distributed. Due to the hydrophilic property and three-dimensional channel network with an asymmetrical aperture, the zeolite filled NaAlg membrane performed higher water sorption and selectivity. As a result, the MMM exhibited both enhanced water permeation flux and a higher separation factor for ethanol dehydration. Xue et al. improved the throughput of a PDMS based membrane by imbedding CNTs into the polymer matrix [108]. Owing to the fast ethanol transport through the inner tubes or along the smooth surface of the CNTs, the ethanol flux in permeate was increased by 10 times without sacrificing membrane separation efficiency when the ethanol content in the feed solution was increased from 1% to 30% (v/v).

## 4. Application of Nanofiller MMMs for PV Processes

In general, PV MMMs involve hydrophilic and organophilic MMMs depending on the host polymer characteristics and there are two general types of MMMs—namely composite membrane and free-standing membrane. When applied in a PV process, the free-standing or the active layer of the composite membrane is directly contacted with the feed (liquid mixture). By maintaining a vacuum or lower partial vapor pressure than the liquid feed mixture on the other side of the membrane, the penetrants are continuously removed as a vapor and finally trapped in a condenser. Alternatively, a sweep or carrier gas can also be applied at the permeate side of the membrane to facilitate the PV separation [109]. Specially, hydrophilic PV MMMs are suitable for the selective separation of water from mixtures containing organic solvents or ions. Conversely, the removal of organics from water or the separation of specific organic from organic–organic mixtures is usually performed using organophilic PV MMMs. Since the launch of the commercialized PVA/PAN composite membrane for ethanol dehydration in the 1980s by GFT, the industrialization of PV and research of new materials for PV membranes have extensively increased. At present, PV processes are frequently employed in separating azeotropic and close-boiling point mixtures which are challenging to separate by distillation [110]. Various nanofiller incorporated MMMs have been studied in the aforementioned applications.

### 4.1. Applications of Nanofillers in Hydrophilic PV

#### 4.1.1. Dehydration of Organic Solvents

In chemical industries, organic solvent dehydration (e.g., alcohols [111], ethers [112], hydrocarbon [113], etc.) has been largely conducted via PV processes, which allows for the selective transport of water through hydrophilic membranes while rejecting the organics (retentate). Therefore, that is the major research area for PV membrane applications. Nanomaterials covering all dimensions discussed previously have been investigated for organic solvent dehydration, including silica, TiO_2_, Na^+^-MMT, zeolite, CNTs, ZIFs, GO and MXene as summarized in Table 2. Hydrophilic nanomaterials are favorable since they can be well dispersed in a range of different polymer matrices. The obtained organic–inorganic hybrid membranes exhibit improved physicochemical properties as discussed above and, more importantly, a controlled membrane nanostructure with lower mass transfer resistance.

For example, hydrophilic zeolite 4A was used to tailor the material properties of PVA for ethanol dehydration [114]. The calculated Arrhenius activation energy of water transport was found to be significantly reduced with a concurrent higher energy barrier inhibiting ethanol from permeating through the membrane. As a result, enhanced water flux and a higher separation factor were achieved, showing the feasibility of zeolite enhanced MMMs in application of alcohol dehydration. Xu et al. prepared an MXene/CS MMM for the dehydration of various organic solvents, including ethanol, ethyl acetate and dimethyl carbonate [50]. The formation of assembled MXene nanosheets in the polymer matrix provided water transport channels while restraining the water sorption of the membrane. The resultant MMM exhibited a higher total flux and/or separation factor than the most polymer-based membranes and it also showed comparable performance with two-dimensional laminar membranes in solvent dehydration. The addition of UiO-66 into 6FDA-HAB/DABA polyimide with 30 wt% loading resulted in enlarged free-volume radius and fractional free volume as confirmed by positron annihilation lifetime spectroscopy [78]. The molecule sieving ability of UiO-66 both favored water permeation and maintained the high selectivity of the membrane. The PV dehydration of ethanol, isopropanol and n-butanol was demonstrated with higher water flux and separation factors than other state-of-the-art polymeric PV membranes. GO nanosheets were grafted with benzenesulfonic acid to enhance their hydrophilicity. The resultant benzenesulfonic acid grafted GO produced more defects that allowed non-selective permeation of water. The fabricated MMM containing NaAlg as the polymer matrix and benzenesulfonic acid grafted GO (1.5 wt% with respect to NaAlg) as the nanofiller exhibited 2.4 times greater water flux and 6.6 times greater separation factor compared with the neat NaAlg membrane for PV of 90 wt% ethanol/water mixture at 70 °C [115].

#### 4.1.2. Desalination

Desalination by PV has been the subject of increased research interest as an emerging area in recent years. The first study can be dated back to the 1990s when sulfonated polyethylene hollow fibers were used as a PV membrane in a water desalination system [120]. In the 2000s, polymeric membranes, including cellulose [121], polyester [122], polyether amide [123] and inorganic membranes, including zeolite [124], carbon template silica [125], hydroxyl sodalite [126], and fluoroalkylsilane-ceramic [127] were investigated for PV desalination process with high salt rejections of above 99% and fluxes of 6.7 (cellulose, 40 °C, 20 Pa), 0.56 (polyether amide, 70 °C, cooler tunnel), 7.1 × 10^−3^ (polyester, 20 °C, the membrane tube placed in sand), 2.2 (carbon template silica, 25 °C, vacuum), 3.9 (hydroxyl sodalite, 200 °C, 300 Pa) and 5 kg m^-2^ h^-1^ (fluoroalkylsilane-ceramic, 40 °C, 400 Pa). Over the past 10 years, continuous progress on the development of organic and inorganic PV desalination membranes was achieved. Membranes fabricated by stacking two-dimensional nanosheets, such as GO or MXene nanosheets, also exhibited high separation performances in PV desalination [14,128]. Meanwhile, MMMs began to attract attention with particular focus on the effect of nanofillers on the desalination performance, which is summarized in Table 3.

Mizsey and coworkers employed Laponite XLG nanoclay as nanofiller to produce dense PVA based MMMs [129]. The hydrophilic Laponite nanoclay increased the PVA surface hydrophilicity and mechanical stability. More importantly, the salt permeability was significantly reduced upon the addition of Laponite, which turned out to be only one hundredth compared with water permeability. As a result, the MMM with 2 wt% Laponite XLG loading exhibited a high-water flux of 58.6 kg/m^2^ h and a salt rejection of over 99.9% at 70 °C using aqurous NaCl as the feed (3 wt%). Xie et al. investigated the effect of operating conditions on the desalination performance of PVA/silica MMM [104]. The salt rejection was found to be independent of various operating conditions, including feed temperatures, flow rates and concentrations, as well as permeate pressures due to the non-volatile nature of NaCl. In addition, the water transport through the membrane was shown to be unaffected by the feed flow rate since the diffusion of water within the membrane was the rate controlling the step rather than the adsorption of water on the membrane surface. However, the salt concentration changed the activation energy of water permeation, thus showing increased water permeation flux when the salt concentration was reduced.

### 4.2. Applications of Nanofillers in Organophilic PV

#### 4.2.1. Removal of Organic Solvents from Water

Compared with conventional distillation, adsorption and extraction approaches, organophilic PV is reported to be more economically feasible for the production of biofuels and bio-based chemicals [132]. As for the membrane materials, several polymers, including polyurethane, PEBA, nitrile-butadiene rubber, styrene-butadiene rubber and PDMS have been used as the membrane materials in various attempts of the removal of organics from water [132,133,134]. Among them, PDMS is the dominant material for organophilic PV and it is widely applied in the separation of ethanol, chloroform, benzene, styrene, etc. from water with good long-term stability. However, PDMS membrane suffers from limited selectivity and low organic flux. Endeavor is mainly devoted to the selection of nanofillers for PDMS based MMMs to pursue both enhanced organic permeation and selectivity. 

To date, fumed silica, zeolite (ZSM-5), silicalite (silicalite-1 and hollow silicalite spheres), COF and MOF (MAF-6 as well as ZIF-7, 8, 67, 71, 90) have been investigated as nanofillers in PDMS based MMMs for enhanced hydrophilic PV [53,90,135,136,137,138,139,140,141,142]. A summary of some typical nanofillers used in this respect are shown in Table 4. For instance, Qin and coworkers enabled both increments in organic transport and the selectivity of furfural over water in PDMS based MMM via doping porous nanofillers (COF-300) that have ultrahigh affinity with the organic solvent [143]. The presence of hydrophilic amino and aldehyde groups in the pore channels of COF-300 formed hydrogen bonding interaction sites, which effectively impeded water transport. Consequently, when separating a 1.0 wt% furfural aqueous solution at 80 °C, the 8 wt% COF-300/PDMS MMM showed a 42.7% increase in furfural selectivity and a 14.1% increase in furfural permeability with respect to the neat PDMS membrane. In addition to the PDMS based MMMs, surface-modified silica NPs using hexadecyltrimethylammonium have been blended with PTMSP recently, with the hydrophobicity of the membrane improved when compared to the neat PTMSP [40]. The resulting surface-modified silica remarkably enhanced the diffusion of 1-butanol, giving rise to a separation factor of 126 and total flux of 1.74 mg cm^−2^ min^−1^ at 63 °C (1.5 w/w% 1-butanol in the feed). GO has also been added into polymer of intrinsic microporosity, PIM-1, for the recovery of 1-butanol [144]. In this study, the incorporation of GO at only 0.1 wt% resulted in more than double the separation factor compared with the pure PIM-1 membrane while maintaining the permeation flux. In addition, 2-phenylethyl group modified silicalite-1 was also incorporated into PIM-1 for removal of ethanol from water. The resulting MMM exhibited higher ethanol selectivity as well as CO2/N2 selectivity with reduced membrane permeability than the pristine PIM-1 membrane [145].

#### 4.2.2. Separation of Organics from Organic Mixtures

PV of organic/organic mixtures mainly involves the separation of polar/non-polar solvent mixtures, aromatic/alicyclic mixtures, isomer mixtures and aromatic/aliphatic hydrocarbon mixtures. The first commercial organic–organic PV was established by Air Products in the 1990s, with the purpose of separation of methanol from methyl-*tert*-butyl ether (MTBE) [132]. In the early stages of development, hydrophobic materials, such as polyethylene and polypropylene, were mainly used to form membranes for organic/organic PV separations. However, these hydrophobic membranes did not show high selectivity or permeation flux since they do not contain functional groups that are required to impart specific interactions with one of the components in the mixtures for effective separation. Different from the PV in aqueous/organic separation using various hydrophilic materials, the lack of appropriate membrane materials is a long-standing challenge for the further industrialization of organic/organic PV processes. The type of mixtures requires specific membrane chemistry and thus membrane design should be flexible.

MMMs are considered as potential candidate for organic–organic separation due to their diverse combinations of polymer and nanofiller. For example, Hou et al. used the sol-gel method to obtain a TiO_2_ incorporated ethyl cellulose membrane for gasoline desulfurization [148]. The resulting MMM with 10 wt% TiO_2_ content exhibited a doubling of flux with only a slight decrease in the sulfur enrichment factor (from 3.69 to 3.13). Jiang and coworkers dispersed CNTs in PVA with the assistance of β-cyclodextrin [149]. The MMM was applied in the separation of benzene/cyclohexane (50/50 wt.%) mixtures. Owing to the π–π interaction between CNT and benzene molecules, the sorption selectivity of the membrane was enhanced after the incorporation of CNTs. The best separation performance was obtained with a benzene permeation flux of 42.3 g m^−2^ h^−1^ and separation factor of 36.4 when the CNT content was 6 wt% with respect to PVA. A MOF Cu_3_(BTC)_2_ incorporated PDMS membrane was adopted for the selective transport of thiophene [150]. The Cu_3_(BTC)_2_ provided multifunctions during the separation, including the transport sites compromising the active metal sites, molecule sieve channels and disordered PDMS chain packing, which were all beneficial for the fast separation of thiophene from the feed. When Cu_3_(BTC)_2_ was 8 wt% of PDMS, the permeation flux and enrichment factor were increased by 100% and 75% in comparison with the pure PDMS membrane, respectively. In addition, MIL-101 was also added into PDMS successfully to enhance the separation performance in the desulfurization of model gasoline due to the increased free volume, intrinsic nanochannels from MIL-101 and adsorption selectivity of MIL-101 for thiophene. The resulting MMM with 6 wt% MIL-101 loading exhibited remarkable 236% flux and 138% enrichment factor of the control PDMS membrane [151]. More of the MMMs used in separations of organics from organic mixtures are tabulated in Table 5.

## 5. Conclusions and Future Outlook

With the rapid development of nanomaterials over the last two decades, MMMs have made great progress in PV applications. This review summarizes the nanomaterials that have been selected as the nanofiller in terms of the dimensions. Generally, zero-, one-, two- and three-dimensional nanomaterials have been actively embedded into various polymer matrices for the purpose of obtaining high-performance separation applications by PV. These applications include organic solvent dehydration, desalination, and separation of organics from organic–organic or organic–aqueous mixtures. Various combinations of nanofillers and polymers provide unique and favorable physicochemical properties, including surface hydrophilicity or hydrophobicity, thermal stability, tuned free volume, varied crystallinity and chemical functionalities, which underpinned the selectivity and permeability of the MMMs. To date, however, knowledge of whether the nanofillers are dispersed molecularly or in a semi-aggregated state is not well reported and needs further in-depth investigation. The transport of solutes in the inorganic dispersed phase and organic–inorganic interphase are also not fully understood. Therefore, there remains rich opportunities for a new understanding of the dispersion of nanofillers and molecule transport with the advancement of material characterization technologies and computational simulation tools. In addition, the future growth of PV processes will be strongly related to the development of novel membrane materials, including the large-scale industrial fabrication of MMMs, as well as the scalable preparation of novel nanomaterials, which remains a challenge. Novel approaches of new MMMs with strong resistance to harsh chemical compounds and high temperatures can also encourage and advance the use of PV in new application fields. Efforts are continuously needed to explore the great potential of MMMs and scalable fabrication techniques to further improve PV separation performance, thus making PV more attractive and commercially viable in practical applications.

## Figures and Tables

**Table 1:** Typical nanofillers of different dimensions in polymer matrices for pervaporation.

Nanofiller	Polymer Matrix	Dimension	Size (nm)	Application (A/B Separation)	T (°C)	Flux (g m^−2^ h^−1^)	Separation Factor (A/B)/Salt Rejection	Ref.
Silica	PVA	0	<10	Desalination	22	6930	99.5 (%)	[24]
CTAB-silica	PTMSP	0	-	Butanol/water	63	1044	126	[40]
TiO_2_	CS	0	100	Water/ethanol	80	340	196	[26]
Ag	Nafion	0	-	Benzene/cyclohexane	25	1.6	12.65	[41]
GQDs	Alg	0	<20	Water/methanol	70	2323	29.5	[42]
GOQDs	NaAlg	0	3.9	Water/ethanol	76	2432	1152	[43]
CNT	PVA	1	Length: 500–2000 Outer diameter: < 8	Desalination	55	11,860	99.9 (%)	[44]
Fe_3_O_4_/CNT	NaAlg	1	Fe_3_O_4_: 10 CNT diameter: 20–30 CNT length: -	Water/ethanol	76	2211	1870	[45]
Attapulgite nanorods	NaAlg	1	Length: 300–1000 Outer diameter: 20	Water/ethanol	76	1356	2030	[46]
Titanate nanotubes	PVA	1	Length: 100–200 Outer diameter: 10-20	Water/isopropanol	50	~30	5520	[47]
Aluminosilicate nanotubes	PVA	1	Length: ~500 Outer diameter: ~2.2	Water/ethanol	60	333	-	[48]
GO	polyimide	2	Lateral size: <1000 Thickness: <2	Water/isopropanol	60	161.5	>5000	[49]
MXene	CS	2	Lateral size: 500–1000 Thickness: 1–2	Water/ethanol	50	1424	1421	[50]
g-C_3_N_4_	NaAlg	2	Lateral size: - Thickness: ~0.96	Water/ethanol	76	2469	1635	[51]
MoS_2_	Pebax	2	Lateral size: 1000–2000 Thickness: 6	Thiophene/n-octane	60	11,420	-	[52]
ZSM-5	PDMS	3	4900	Ethanol/water	40	408	14	[53]
zeolite 4A	polyimide	3	300–400	Water/isopropanol	30	18	8991	[54]
ZIF-71	PEBA	3	1000	Butanol/acetone–ethanol–water	37	96.8	18.8	[55]
Na^+^-MMT	PVA	3	800	Water/isopropanol	30	51	1116	[56]
CMS	PDMS	3	<50,000	Benzene/water	40	~140	9000	[57]
GOF	PVA	3	Lateral size: - Thickness: 6.5–9.1	Water/ethanol	70	~300	330	[58]

**Table 2:** Typical nanofillers used in dehydration of solvent.

Nanofiller	Polymer Matrix	Solvent	Water in Feed (wt%)	T (°C)	Flux (g m^−2^ h^−1^)	Separation Factor	Ref.
NH_2_-MIL-125	NaAlg	Acetic acid	10	30	196.7	328.1	[116]
rGO/GQD	Alg	Methanol	30	70	2323	29.5	[42]
zeolite 4A	PVA	Ethanol	23.57	60	936	710	[114]
Fe_3_O_4_/CNT	NaAlg	Ethanol	10	76	2211	1870	[45]
Attapulgite nanorods	NaAlg	Ethanol	10	76	1356	2030	[46]
GOQDs	NaAlg	Ethanol	10	76	2432	1152	[43]
NaA zeolite	Poly(acrylic acid) sodium	Ethanol	10	30	533.2	435.7	[117]
g-C_3_N_4_	NaAlg	Ethanol	10	76	2469	1653	[51]
Cu_3_(BTC)_2_	Polyimide	Ethanol	10	42	430	~200	[118]
MXene	CS	Ethyl acetate	2	50	1471	4898	[50]
UiO-66	Polyimide	Isopropanol	15	60	225.9	2209	[78]
GO	Polyamide	Isopropanol	10	70	6593	1491	[74]
ZIF-8	PVA	Isopropanol	10	30	952	91	[100]
GO	Polyetherimide	Butanol	5	50	1100.26	89.39	[75]
Fe_3_O_4_	PVA	Tetrahydrofuran	5	30	95	519	[119]

**Table 3:** Nanofillers used in PV desalination.

Nanofiller	Polymer Matrix	NaCl in Feed (wt%)	T (°C)	Flux (g m^−2^ h^−1^)	Salt Rejection (%)	Ref.
GO	CS	3.5	60	17,700	99.9	[23]
GO	polyimide	3.5	90	15,600	99.8	[130]
GO	PVA	10	65	28,000	99.9	[131]
silica	PVA	0.2	22	6930	99.5	[24]
CNT	PVA	3.5	55	11,860	99.9	[44]
Laponite	PVA	3	60	51,200	99.9	[129]

**Table 4:** Typical nanofillers used in PV separations of organics from organic–aqueous mixtures.

Nanofiller	Polymer Matrix	Permeate	Water in Feed (wt%)	T (°C)	Flux (g m^−2^ h^−1^)	Separation Factor	Ref.
ZSM-5	PDMS	Ethanol	95	40	408	14	[53]
CMS	PDMS	Benzene	99.95	45	145	11,750	[57]
MAF-6	PDMS	Ethanol	95	40	1200	14.9	[90]
COF-300	PDMS	Furfural	95	80	2136	39.6	[143]
ZIF-90	PDMS	Ethanol	95	60	99.5	15.1	[142]
MIL-53	PDMS	Ethanol	95	70	5467	11.1	[146]
Silica	PTMSP	Butanol	98.5	63	165	126	[40]
rGO	PIM-1	Butanol	95	65	649.7	27.1	[144]
ZIF-71	PEBA	Butanol	98.8	37	96.8	18.8	[55]
POSS	Pebax	Ethanol	95	25	183.5	4.6	[147]

**Table 5:** Other MMMs used in PV separations of organics from organic mixtures.

Nanofiller	Polymer Matrix	Permeate	Feed	T (°C)	Flux (g m^−2^ h^−1^)	Separation Factor	Ref.
POSS	PDMS	Benzene	Benzene/n-heptane	70	~82	~3.5	[152]
POSS	PDMS	Thiophene	Thiophene /n-heptane	70	~125	~4.2	[152]
POSS	PDMS	Toluene	Toluene/n-heptane	70	~70	~3.3	[152]
Al_2_O_3_	Polyamide	Methanol	Methanol/MTBE	30	476	20	[153]
Ag-GO	Polyimide	Benzene	Benzene/cyclohexane	30	1560	35	[154]
Cu_3_(BTC)_2_	PVA	Toluene	Toluene/n-heptane	40	133	17.9	[155]
Zeolite	PVA	Methanol	Methanol/benzene	30	71.03	47	[156]
Silicalite	CS	Toluene	Toluene/methanol	30	19	264	[157]
GO	PVA	Toluene	Toluene/n-heptane	40	27	12.9	[158]
Zeolite	Polyvinyl chloride	Benzene	Benzene/cyclohexane	80	329.7	8.04	[159]
Ag/CNT	Polyurethane	Benzene	Benzene/cyclohexane	30	2375	64.8	[66]
